# Does the healthy immigrant effect apply to mental health? Examining the effects of immigrant generation and racial and ethnic background among Australian adults

**DOI:** 10.1016/j.ssmph.2018.10.011

**Published:** 2018-10-25

**Authors:** Rennie Lee

**Affiliations:** University of Melbourne, School of Social and Political Sciences, John Medley (Building 191), Parkville, VIC 3010, Australia

**Keywords:** Healthy immigrant effect, Australia, mental health, children of immigrants, race and ethnicity

## Abstract

The healthy immigrant effect (HIE) refers to the phenomena in which immigrants show greater health outcomes than the native-born population. However, it is unclear what is the extent to which HIE applies to various outcomes and populations. Much of the work on HIE has revolved around physical health outcomes; mental health, however, has not garnered the same level of attention with regard to HIE. It is also uncertain whether immigrants’ health advantage persists beyond one generation. This study assesses the mental health of the first, second, and third and higher generations (70,517 person-year observations) for individuals from various racial and ethnic backgrounds in Australia using Waves 1−16 of the Household Income and Labour Dynamics in Australia survey. The dependent variable is mental health score and key independent variables include generation and racial and ethnic background. I control for age, educational attainment, labor force status, marital status, remoteness, household income, language, neighborhood disadvantage, citizenship, weight, and gender. Using linear regression with random effects, this study finds that mental health varies by generation; the third and higher generation show the greatest mental health score, followed by the first generation and the second generation, net of controls. Mental health score also varies by racial and ethnic background. Except for English-speaking groups, native-born Australians show a clear advantage over Europeans, North Africans/Middle Easterners, and Asians. Racial and ethnic disparities differ by generation and are strongest among the first generation. My findings extend HIE, which typically emphasize immigrants’ superior health outcomes over the native-born population but do not focus on racial and ethnic disparities among immigrants. My results suggest that immigrant groups vary widely in their mental health outcomes but these lessen over time. Overall, the findings suggest the limited applicability of HIE for a broad range of health outcomes and populations.

## Highlights

• The third and higher generation have the highest mental health scores, followed by the first and second generations.
• The first generation's mental health scores are associated with relatively fewer factors relative to the second and third and higher generations.
• English-speaking groups show the highest mental health followed by native-born Australians.
• All other minority groups show lower mental health scores.
• Racial and ethnic disparities in mental health differ by generation.

## Introduction

There is growing international focus on mental health illnesses as a public health priority, given its association with worse physical health, decreased productivity, and premature death (Judd & Humphreys, 2001). The risk of mental health issues, however, are not equally distributed across the population. Immigrant and minority communities, in particular, are at risk; nearly 15% of foreign-born individuals in Australia experience some kind of mental disorder (Minas et al., 2013). The reasons range from receiving lower access to care, experiencing discrimination in the host society, and facing migration-related challenges (Minas et al., 2013; Schweitzer, Brough, Vromans & Asic-Kobe, 2011). Understanding the mental health issues of these communities is especially salient in Australia, where nearly half of the population is an immigrant or a child of an immigrant (Australian Bureau of Statistics, 2013, Australian Bureau of Statistics, 2016). Continuous migration remains a primary driver of population growth in Australia so the mental health outcomes of these communities have tremendous long-term social, economic, and health consequences (Spinks & Koleth, 2010).

## Healthy immigrant effect

A large literature on immigrant health has focused on the healthy immigrant effect (HIE), which refers to the phenomena in which immigrants in industrialized nations show greater health outcomes than native-borns in the host country (Urquia, O’Campo, & Heaman, 2012; Wu & Schimmele, 2005). This includes self-rated health, mortality, reproductive health, BMI, and to a lesser extent mental illness, across several national contexts (Biddle, Kennedy & McDonald, 2007; Cunningham, Ruben, & Venkat Narayan, 2008; Menigoz, Nathan, & Turrell, 2016; Noymer & Lee, 2012; Urquia et al., 2012). Immigrants’ greater health outcomes are paradoxical since they typically have fewer financial resources, less advantages, and occupy lower positions in the social hierarchy than native-borns (Urquia et al., 2012). Such disadvantages and the social stress associated with migration and adapting to a new host population would suggest worse physical and mental health (Wu & Schimmele, 2005).

A majority of work supporting HIE has revolved around physical health outcomes (e.g., cancer, cardiovascular disorder, disabilities, etc.) (Commodore-Mensah et al., 2018, Monserud, 2017; review in Wu & Schimmele, 2005). Mental health, however, has not garnered the same level of attention with regard to HIE and studies focusing on the two together show conflicting results (Ali, McDermott, & Gravel, 2004; Missinne & Bracke, 2010; Straiton, Grant, Winefield & Taylor, 2014). Specifically, there is less consensus on whether the relationship between migrant status and mental health is positive or negative. Thus, this study extends the findings of HIE in several ways by examining the mental health outcomes of immigrants and their descendants.

HIE has been attributed to general explanations related to selection, acculturation, and discrimination. While these explanations emphasize immigrants’ health, they are ambiguous about the health of immigrants’ children and racial and ethnic minorities. There is some recognition that immigrants’ health advantage fades over time, but it is uncertain whether this occurs within one generation or across several generations (Acevedo-Garcia, Bates, Osypuk & McArdle, 2010). Moreover, it is unclear whether health advantages remain as immigrants become racial and ethnic minorities in the host society. The effects of selection, acculturation, and discrimination are long-standing though and are likely to affect subsequent generations (Portes and Rumbaut, 2001, Telles and Ortiz, 2009).

This study expands on these explanations that primarily focus on immigrants to assess the mental health outcomes of the first generation (foreign-born individuals), second generation (native-born children of immigrants), and third and higher generation (native-born children of native-born parents). Whereas most assessments of HIE compare foreign-borns and native-borns across several country contexts (Kennedy et al., 2015, Menigoz et al., 2016, Noymer and Lee, 2012, Urquia et al., 2012), this study includes a generational distinction because a large body of sociological research indicates that the second generation have different outcomes than the third and higher generation (Luthra & Waldinger, 2013; Portes & Zhou, 1993). Moreover, most explanations for HIE discuss changes over time, but have not assessed it using a generational framework. Instead, this study uses generation status as a marker of time to assess health changes over time. I use the third and higher generation to represent the host population. Understanding the extent to which HIE applies to various populations or subgroups is important because if HIE is only applicable to certain segments of the population, it is not a reliable general concept (Wu & Schimmele, 2005).

## Health outcomes across generation

The first perspective, immigrant selection hypothesis, emphasizes the effects of immigrants’ premigration characteristics and immigrant selection on health outcomes. Immigrants may be selective relative to those left behind in the origin country, but they can also be selective compared to the general population in the host country. In regard to HIE, the focus has been on the latter with results showing immigrants’ superior health outcomes relative to native-borns of the host country.

Additionally, immigrants may be selected via several processes, which are complex and interrelated. They may be self-selected since individuals with greater health in the origin country are more likely to migrate (Escarce et al., 2006). For instance, individuals who migrate may be more likely to take chances and navigate difficult circumstances (Gong, Xu, Fujishiro & Takeuchi, 2011; Kuo & Tsai, 1986; Wu & Schimmele, 2005). Immigrants can also be selected by host countries vis-à-vis national immigration policies, such as points systems that can screen for healthier migrants based on characteristics like age, education, language, and financial resources (Kennedy, McDonald & Biddle, 2006). As a result of various selection processes, immigrants have greater premigration characteristics and possess other psychological resources or personality traits, such as initiative and the motivation to succeed, which are conducive to health (Mossakowski, 2007). For instance, economic migrants selected on their education, language, or skills may have greater labor market success, which enhances and protects mental and physical health (Newbold, 2009). Selective migrants with these personal and psychological resources may have a greater capacity to cope with various kinds of social stress, which decreases the likelihood of depression (Wu & Schimmele, 2005). This is advantageous for their children, who experience less familial discord, greater parent-child interaction, and greater physical and mental health throughout their life (Pilowsky, Wickramaratne, Nomura & Weissman, 2006; Weissman et al., 2006). Overall, this perspective posits that the first and second generations experience greater health outcomes than the third and higher generations. According to this perspective, the first and second generations are selective groups and thus, experience greater mental health than the host population.

A second perspective, immigrant acculturation hypothesis, posits that new immigrants are healthier because they have not yet adopted the behaviors and norms of the native-born population. Cultural norms, behaviors, and social characteristics operate vis-à-vis health and lifestyle behaviors, family structure, and social networks that provide a protective factor for immigrants (Escarce et al., 2006). For instance, immigrants show lower rates of tobacco, drug, and alcohol use, better nutrition, and greater social and family support (e.g., presence of partners and extended family) than native-borns, all of which contribute to greater mental health (Kennedy et al., 2015; Singh & Siahpush, 2002). As immigrants and their descendants lose attributes of the immigrant culture and adopt those of the host society, they may also relinquish health-promoting behaviors and norms. In turn, their health worsens and converges with native-borns (Escarce et al., 2006; Singh & Siahpush, 2002). For instance, immigrants’ children who are English monolinguals have lower psychological well-being and greater depression because a loss of the immigrant language weakens their connection with social supports, like the immigrant family and the coethnic community (Leong, Park, & Kalibatseva, 2013; Portes & Rumbaut, 2001). This perspective hypothesizes that health status declines with time in the host country. Thus, based on this perspective, the first generation will have the greatest health outcomes followed by the second generation and the third and higher generation.

A third perspective, structural disadvantage hypothesis, emphasizes the role of discrimination or blocked social mobility associated with racial and ethnic minority status. Racial discrimination creates social and economic inequities along racial and ethnic lines, which are directly related to disease (Adler & Rehkopf, 2008; Dolezsar, Jennifer, Herzig & Miller, 2014; Viruell-Fuentes, Miranda & Abdulrahim, 2012). The consequences of belonging to a minority group in the host country can negatively affect health and increase the incidence of depressive symptoms (review in Bernstein, Park, Shin, Cho & Park, 2009; Leonardo, 2016). New immigrants, however, may experience a protective effect since many were not minorities in the origin country and they have not yet been exposed to the detrimental effects of racial discrimination in the host country (Mossakowski, 2007). Nonetheless, it is unclear how long this protective effect may last. Thus, this perspective makes several hypotheses regarding generation and racial and ethnic background. Immigrants will have greater health than native-borns. However, health status differs by racial and ethnic groups and native-born whites will exhibit greater health than native-born minorities (Acevado-Garcia, Bates, Osypuk & McArdle, 2010).

Overall, the three perspectives posit different health outcomes by generation and vary in the extent to which they address mental health outcomes per se. Empirical studies on the effects of nativity and generation on mental health are mixed in regards to whether immigrants and minorities have any mental health advantage over the native-born population (Ali et al., 2004, Beiser et al., 2002; Harker, 2001; Leonardo, 2016; Missinne & Bracke, 2010; Mossakowski, 2007; Straiton et al., 2014). Thus, this study considers how the predictors of mental health compare across generation and racial and ethnic background to elucidate the broader implications of HIE as well as the predictors of mental health.

## Australia’s immigration and health context

Since the mid 1980s, Australia’s immigration policy has increasingly emphasized skill in its selection criteria. Entrants must satisfy a “points test” that selects migrants based on age, education, language, and occupation and also satisfy minimum health requirements (Antecol, Cobb-Clark, and Trejo, 2003; Clarke and Isphording, 2017). Changes in immigration policy have altered the sending countries of incoming immigrants and Australia’s racial and ethnic composition. In the immediate postwar period, most migrants arrived from the United Kingdom, Ireland, and a few European countries (Khoo, Hugo & McDonald, 2008). British migration dominated until about the early 1970s. This coincided with the end of the White Australia policy and the Vietnam War, which increased migration from Asia, the Pacific, Middle East, and Africa (Khoo, McDonald, Giorgas & Birrell, 2002). Asian migration has continued since the late 1960s (Krupinski, 1984). Most non-European migration occurred during the late postwar period (Khoo et al., 2002). Overall, Australia’s selective migration policy and racial and ethnic diversity make it an excellent case for understanding health outcomes of immigrants, their children, and racial and ethnic minorities.

## Data and methods

This study analyzes annual panel data from 2001−2016 Household Income and Labour Dynamics in Australia (HILDA) survey. HILDA is a household panel study that collects information on physical and mental health, education, income, race and ethnicity, and migration background. HILDA interviews all individuals aged 15 or older in the household; new participants are added if they join the household or turn 15 while living in the household. In Wave 11, there was a top up of 2153 households, increasing the overall number of immigrants and racial and ethnic minorities (Menigoz et al., 2016). This study analyzes the data using linear regression analyses with random effects.

### Analytic sample

This study focuses on the first, second, and third and higher generation individuals aged 18 and older. The first generation represent foreign-born individuals who arrived at age 15 or older; the second generation includes native-born individuals with at least one foreign-born parent and foreign-born individuals who arrived at age 14 or younger; and the third and higher generation consist of native-born individuals with native-born parents. I include foreign-born individuals who arrived before age 15 (1.5 generation) with the second generation because they share similar experiences of socialization, schooling, and language (Portes & Zhou, 1993). Nearly 75% of the 1.5 generation in my sample arrived before age 9, so most are socialized in Australia. Racial and ethnic background is assigned to first and second generation individuals based on mother’s country of birth. If this is unavailable, respondents are assigned their father’s country of birth. Third and higher generation individuals are coded as native-born Australians with Australian-born parents. Contemporary migrant groups in Australia have small populations of third generation individuals. There are some third generation British, but it is not possible to decipher this information from the data. Aside from third and higher generation Australians, all other racial and ethnic groups are first or second generation. Thus, the racial and ethnic analyses make distinctions between ethnic-immigrant groups versus Australian-borns. I started with 317,738 person-year observations. There are two analytic samples focusing on racial and ethnic groups (70,517 person-year observations) and generation status (86,312 person-year observations), which are derived after including variables of interest and implausible and missing data are removed.

### Dependent variable

The dependent variable is the Mental Component Summary (MCS) score. It is drawn from the Short Form 36 (SF-36), a widely-used and reliable measure of health status (Butterworth & Crosier, 2004). The MCS score is a psychometrically-based measure that is calculated from several subscales capturing the role limitations caused by mental health, emotional problems, social functioning and vitality. The MCS score ranges from 0 to 100; a higher score represents greater mental health.

### Key independent variables

The key independent variables are generation and racial and ethnic background. Generation is a categorical variable: first generation, second generation, and third and higher generation. Region of origin is a categorical variable: English-speaking, European, North African and Middle Eastern, Asian, native-born Australian, and other.^1^

### Control variables

This study includes control variables for demographic characteristics, such as age (average age around 46 years old) and female versus male (reference category), and other individual characteristics, such as educational attainment (diploma or certificate, bachelor’s degree, graduate/postgraduate degree, and high school degree or less as the reference category), labor force status (unemployed, not in labor force, and employed as the reference category), marital status (married, widowed, single, and divorced/separated as the reference category), household income (20,000–39,999, 40–59,999, 60,000–79,000, 80,000–124,999, 125,000+, and 0–19,999 as the reference category), monolingual versus bilingual or more (reference category), citizen versus not (reference category), years in Australia (less than 5 years, 5–9 years, 10–14 years, and 15 years or more as the reference category), and overweight (a body mass index exceeding 25 kg m^−2^) versus not (reference category) (World Health Organization, 2018). The analyses also control for contextual characteristics, such as area remoteness (rural, remote, and urban as the reference category) and neighborhood disadvantage (measured as quintiles with the poorest quintile as the reference category). Summary statistics are presented in Appendix A Table A1 for the first, second, and third and higher generations.

## Statistical analyses

The analytic strategy consists of a series of random-effect linear regression models to assess the associations between mental health and (a) racial and ethnic background; and (b) generation status. I use repeated measures of individual’s mental health scores over 16 years so I include random-effects with robust estimators of variance to account for the correlation between individual’s responses and the correlational nature of the data. The logic of the analyses is to first show the extent of racial and ethnic differences in mental health regardless of generation using descriptive statistics and regression models. I show the association between mental health and racial and ethnic background with and without control variables. Then, I assess whether racial and ethnic differences in mental health are mediated by generation status using random-effect linear regression models, net of controls. I run separate models for the first, second, and third and higher generations to show how the relationship between mental health and ethnic background varies for each generation. Additionally, to understand the impact of these variables, I present predicted mental health scores by generation with control variables held at their mean. The data were analyzed with Stata 15.

## Results

**Fig. 1:**
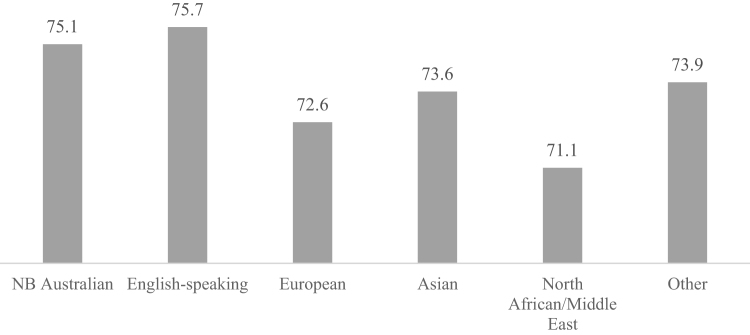
Mental health scores by racial/ethnic background.

Fig. 1 presents the bivariate relationship between mental health and racial and ethnic background without any controls. English-speaking groups (75.7) have the highest scores, followed by native-born Australians (75.1), Asians (73.6), Europeans (72.6), and North African/Middle Eastern (71.1). Thus, Fig. 1 indicates that most ethnic groups experience lower mental health scores relative to native-born Australians, with the exception of English-speaking groups.

### Linear regression with random effects

Table 1 presents the coefficients from a linear regression with random-effects predicting mental health for English-speaking groups, Europeans, Asians, North African and Middle Eastern, Others, and native-born Australians as the reference group. Table 1, Model 1 is the baseline model, showing the relationship between race and ethnicity and mental health without any controls. The coefficient for English-speaking migrants is 0.62 (0.07, 1.18) but not significant. The coefficient for European is −2.43 (−3.16, −1.71) and significant. This shows that Europeans have a lower mental health score compared with native-born Australians. The coefficients for North African/Middle Eastern (−3.94) (−5.89, −2.00) and Asian (−1.13) (−1.98, −0.28) are also significant. These confirm the results in Fig. 1 indicating superior health among native-born Australians.

**Table 1:** Coefficients of random effects predicting mental health score for ethnic groups.

	Model 1	95% CI	Model 2	95% CI
Ethnicity				
English-speaking	0.62*	(0.07, 1.18)	0.21	(−0.34, 0.75)
European	−2.43***	(−3.16, −1.71)	−2.37***	(−3.12, –1.61)
North African and Middle Eastern	−3.94***	(−5.89, −2.00)	−2.61*	(−4.74, −0.48)
Asian	−1.13**	(−1.98, −0.28)	−0.50	(−1.41, 0.42)
Other	−0.39	(−1.63, 0.85)	−0.03	(−1.19, 1.13)
				
(ref: 3+ generation Australians)				
N (observations)	70517		70517	

***P < .001 **p < .01 *p < .05 +*p* < .1 (two tailed tests).

Note: Model 2 controls for age, educational attainment, labor force status, marital status, area remoteness, household income, neighborhood disadvantage, citizenship, gender, weight status, and monolingualism.

Table 1, Model 2 controls for age, educational attainment, labor force status, marital status, area remoteness, household income, neighborhood disadvantage, citizenship, gender, weight status, and monolingualism; I present the coefficients for ethnicity for brevity. Net of controls, European and North African and Middle Easterners show a clear disadvantage relative to native-born Australians, but the difference between Asians relative to native-born Australians is diminished. Overall, Table 1 shows that ethnic disparities in mental health are partly attenuated by controls.

In Table 2, I consider generation status, which may mediate ethnic differences in mental health. The first column shows the association between mental health and ethnic background for the first generation. The coefficient for European is −5.24 (−7.08, −3.39) and borders significance, showing a lower mental health score for European immigrants than English-speaking immigrants by 5.24 points, net of controls. Similarly, the coefficient for North African/Middle Eastern immigrants is −6.43 (−9.83, −3.02), indicating a lower mental health score among European immigrants relative to English-speaking immigrants. All immigrant groups show lower mental health scores than native-born Australians, net of controls. Among the first generation, individuals who are more educated, married or widowed, monolingual, and living in higher quality neighborhoods have greater mental health scores. In contrast, being unemployed and not in labor force is associated with a lower mental health score.

**Table 2:** Coefficients of random effects predicting mental health score by generation status.

	First generation	95% CI	Second generation	95% CI	Third and higher generation	95% CI
Ethnicity						
European	−5.24***	(−7.08, −3.39)	−1.71**	(−2.67, −0.74)	–	
North African and Middle Eastern	−6.43***	(−9.83, −3.02)	−0.54	(−3.31, 2.23)	–	
Asian	−1.35+	(−2.92, 0.22)	−1.26+	(−2.66, 0.14)	–	
Other	−1.37	(−3.25, 0.51)	0.31	(−1.32, 1.94)	–	
						
(ref: English-speaking)						
						
Age categories						
25–34	−1.81	(−4.22, 0.61)	−0.28	(−1.21, 0.66)	−0.76*	(−1.36, −0.17)
35–44	−2.50+	(−5.06, 0.06)	−0.14	(−1.31, 1.03)	−0.94*	(−1.68, −0.19)
45–54	−2.07	(−4.69, 0.54)	−0.29	(−1.58, 1.0)	−0.29	(−1.08, 0.50)
55–64	0.02	(−2.64, 2.68)	2.51**	(1.07, 3.95)	1.87***	(1.05, 2.70)
65–75	2.07	(−0.73, 4.88)	5.41***	(3.8, 7.02)	4.85***	(3.93, 5.76)
75+	2.51	(−0.53, 5.55)	6.55***	(4.61, 8.49)	5.31***	(4.19, 6.43)
						
(ref: 18–24)						
						
Educational attainment						
Diploma or certificate	2.64***	(1.26, 4.01)	0.06	(-0.81, 0.93)	0.52+	(-0.01, 1.05)
Bachelor	2.87***	(1.37, 4.36)	0.42	(-0.61, 1.45)	1.51***	(0.78, 2.23)
Graduate/postgrad	2.68**	(1.08, 4.28)	1.40*	(0.20, 2.61)	1.51***	(0.67, 2.36)
						
(ref: HS degree or less)						
						
Labor force status						
Unemployed	−2.17*	(−4.23, −0.11)	−1.76**	(−3.06, −0.45)	−2.97***	(−3.75, −2.18)
Not in labor force	−2.68***	(−3.68, −1.68)	−2.50***	(−3.29, −1.71)	−2.48***	(−2.94, −2.02)
						
(ref: Employed)						
						
Marital status						
Married	3.33***	(1.72, 4.93)	1.47*	(0.21, 2.73)	2.16***	(1.41, 2.90)
Widowed	2.12+	(−0.29, 4.54)	1.28	(−0.90, 3.45)	0.47	(−0.86, 1.79)
Single	1.02	(−1.03, 3.06)	−0.13	(−1.59, 1.33)	0.30	(−0.56, 1.17)
						
(ref: Divorced/separated)						
						
Remoteness						
Rural	2.31***	(1.1, 3.51)	0.38	(−0.51, 1.27)	0.59*	(0.09, 1.10)
Remote	5.24**	(1.8, 8.68)	3.33*	(0.75, 5.91)	1.29+	(−0.23, 2.81)
						
(ref: Urban)						
						
Household income						
20,000–39,999	−2.19	(−5.63, 1.26)	2.06+	(−0.33, 4.46)	−1.65*	(−3.08. −0.22)
40,000–59,999	−1.44	(−4.93, 2.06)	2.54*	(0.11, 4.95)	−1.33+	(−2.75, 0.09)
60,000–79,999	−0.58	(−4.09, 2.93)	3.21*	(0.76, 5.67)	−0.76	(−2.18, 0.68)
80,000–124,999	−0.19	(−3.71, 3.32)	4.14**	(1.7, 6.58)	−0.25	(–1.67, 1.18)
125,000+	−0.11	(−3.66, 3.45)	4.44***	(2.01, 6.88)	0.12	(–1.32, 1.55)
						
(ref: 0–19,999)						
						
Neighborhood disadvantage						
2nd Quintile	1.52*	(0.1, 2.94)	0.34	(−0.74, 1.42)	0.74*	(0.13, 1.36)
3rd Quintile	1.97*	(0.49, 3.44)	0.59	(−0.5, 1.67)	1.61***	(0.98, 2.24)
4th Quintile	1.63*	(0.27, 3.0)	0.91+	(−0.12, 1.94)	1.45***	(0.81, 2.09)
Richest	2.34**	(0.9, 3.79)	2.49***	(1.41, 3.56)	2.21***	(1.51, 2.90)
						
(ref: Poorest)						
Citizen	−0.59	(−1.69, 0.51)	0.76	(−1.25, 2.77)	–	
						
(ref: not citizen)						
Female	−1.03+	(−2.11, 0.05)	−1.67***	(−2.48, −0.86)	−2.18***	(−2.7, −1.65)
Overweight	−0.21	(−0.98, 0.56)	−0.37	(−0.93, 0.18)	0.23	(−0.11, 0.56)
						
(ref: Not overweight)						
Monolingual	0.42	(−0.62, 1.47)	0.66	(−0.33 1.65)	0.61	(−1.24, 2.46)
						
(ref: Bilingual or more?)						
N (observations)	10028		19337		56947	

***P < .001 **p < .01 *p < .05 +*p* < .1 (two tailed tests).

Note: All models control for year dummies.

The second column shows that second generation Europeans are significantly disadvantaged relative to their English-speaking counterparts but there is no significant difference among the other groups. Age, educational attainment, marital status, household income, neighborhood disadvantage, and gender are associated with greater mental health among the second generation. The third column shows that among the third and higher generation, age, educational attainment, marriage, area remoteness, household income, and neighborhood disadvantage are positively associated with their mental health whereas unemployment and not being in the labor force, and female are negatively associated with their mental health scores.

Overall, Table 2 indicates that ethnic differences in mental health vary by generation with greater disparities among the first generation. This suggests an interactive effect between ethnicity and immigrant status on mental health that is not captured by ethnicity or generation alone. Related, mental health scores are shaped by different factors depending on generation. The mental health of the second and third and higher generations are both shaped by household income, gender, and age, but these factors have little effect on the mental health scores of the first generation. This suggests that demographic and socioeconomic factors are less predictive for the first generation’s mental health.

**Fig. 2:**
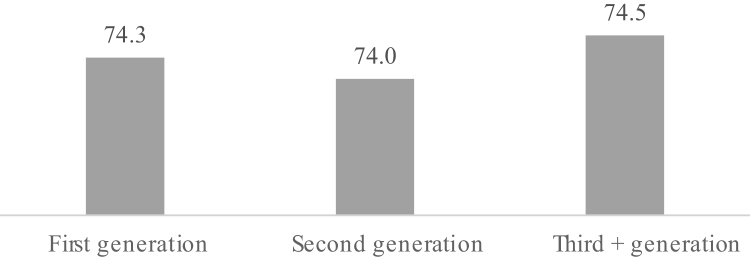
Predicted Mental Health Scores by Generation. Note: Predicted mental health scores control for age, racial/ethnic background, educational attainment, labor force status, marital status, remoteness, household income, language, neighborhood disadvantage, citizenship, weight, and gender.

Fig. 2 presents the predicted mental health scores by generation based on the full model in Table 2, Model 2, with independent variables set at their mean. Fig. 2 shows that net of several demographic, socioeconomic, and contextual characteristics, the third and higher generation still show higher predicted mental health scores (74.5) than the first (74.3) and second generations (74.0).

## Discussion

There are two main findings of this study. First, mental health outcomes vary by generation, net of controls. Descriptively, the third and higher generation shows the highest mental health scores whereas the second generation has the lowest scores, net of controls. The findings suggest that the implications of HIE are exaggerated as they do not extend to mental health outcomes. The first generation are not selective compared to the third and higher generation in regard to their mental health, showing little support for the selection argument in regard to immigrants’ mental health.

My findings contrast with Beiser, Hou, Hyman, and Tousignant (2002), Harker (2001), Kwak (2016), Lau et al. (2013), and Mossakowski (2007), which were based on the US and Canada. One reason my findings differ from Kwak (2016), Lau et al. (2013), and Mossakowski (2007) is because they compared the mental health outcomes of native-borns and foreign-borns, but did not disaggregate by generation. Additionally, the findings may differ from Beiser et al. (2002) and Harker (2001), who examined generation but only focused on young children and adolescents, whose mental health outcomes are shaped by different factors than those of adults (Hoagwood et al. 2001).

Although it is beyond the scope of this paper to identify the mechanisms underlying varied mental health outcomes by generation status, I provide two possible explanations. One reason may be related to Australia’s immigration policy. Australia’s points system can select individuals on characteristics that are associated with physical health, like education, age, language, and country of origin, but these characteristics may not have the same predictive effect for mental health (Chiswick, Lee & Miller, 2008; Landale, Oropesa, & Gorman, 2000). Although higher education is associated with greater physical health vis-à-vis greater occupational status, earnings, resources, and lifestyles, it may not yield the same benefits for immigrants’ mental health. Specifically, immigrants with foreign degrees may have different networks and may not reap the same personal, economic, and social benefits associated with greater mental health, despite their higher educational attainment (Leu et al., 2008). Moreover, Australia’s emphasis on skill-based migration and limited family reunification may negatively affect immigrants’ mental health because they are less likely to have a strong family and social support system, which is associated with greater mental health (cf. Escarce et al., 2006; cf. Ho, 2006). Limited family reunification in migration policies may be particularly harmful for migrants arriving as adults; by comparison, migrants arriving as children or adolescents may not be as adversely affected as they typically migrate with one or both parents. Future research should assess whether Australia’s selective immigration policy adversely affects adult immigrants’ mental health outcomes in ways that it does not for their physical health.

Another potential explanation for diverging mental health scores, specifically the second generation’s poorer mental health, may be related to their unique position that is situated between the immigrant generation and native-born peers (Montazer & Wheaton, 2011). In turn, they may experience multiple reference groups, which can create competing demands from both sides (Portes & Rumbaut, 2001; Zhou, 1997). For instance, Wolf (1997) found that Filipina children of immigrants experienced multiple and contradictory understandings of what it meant to be Filipino, American, and Filipino-American, which were informed by their immigrant families and their own experiences of growing up in the US. These conflicts and internal struggles contributed to their depression and suicidal ideation. Moreover, the second generation may experience discrimination from the host population, which may have a more detrimental effect on their mental health since they are born and/or raised in the host country (Rumbaut, 2005). Thus, the demands associated with navigating two cultures and feelings of not belonging in the immigrant culture or the host population may increase the second generation’s risk of poor mental health (Montazer & Wheaton, 2011; Rumbaut, 2005).

A second major finding is that mental health scores differ by race and ethnicity. With the exception of the English-speaking groups, native-born Australians exhibit higher mental health scores than all other racial and ethnic groups. One reason may be that English-speaking groups share more similar phenotypical, cultural, and linguistic traits with native-born Australians. In contrast, Asians, North Africans, and Middle Easterners are more distinctive and in turn, may encounter more blatant forms of discrimination based on phenotype, culture, and language (Awad, 2010). Cultural similarities with the majority group may facilitate a smoother transition to the host society among European groups (Awad, 2010; Greene, Way, & Pahl, 2006). This is consistent with Araújo and Borrell (2016), Borrell, Catarina, Williams, Diez-Roux & Gordon-Larsen (2006), and Espino and Franz (2002), who found that individuals who share more similar physical features with the majority population, such as skin tone and facial features, perceive and experience less discrimination, which could indirectly affect their mental health.

Related, this study finds that racial and ethnic differences in mental health vary by generation status. In particular, racial and ethnic differences are more evident among the first generation than the second generation. This is consistent with Acevado-Garcia, Bates, Osypuk & McArdle (2010), who found that health outcomes differed by both generation and racial and ethnic background, though they focused on self-rated health. My findings extend other studies of HIE, which typically emphasize immigrants’ superior health outcomes over the native-born population but do not focus on the racial and ethnic disparities within the immigrant generation. My results suggest that immigrant groups vary widely in their mental health outcomes, but these lessen over time. Nonetheless, the results are preliminary; in the absence of longitudinal data spanning longer periods of time, these results should still be interpreted cautiously.

My findings show the most support for the immigrant acculturation hypothesis and structural disadvantage hypothesis, but not the immigrant selection hypothesis. The first generation do not show an advantage in their mental health scores relative to native-born Australians, as suggested by HIE. One caveat is that they exhibit greater mental health scores than the second generation, which may suggest some advantage or protective effect. The findings show that groups that are more similar to the host population in terms of phenotype, norms, and behaviors exhibit greater mental health outcomes. Future research may examine how these interrelated processes work together to shape mental health outcomes.

## Limitations

Although this study was able to assess the mental health scores for a large sample of first, second, and third and higher generation Australians across several racial and ethnic backgrounds, there are some limitations. First, due to the sample size, I cannot examine specific national origin groups. Related, I could not disaggregate the sample by national origin and generation together in a detailed way, which would deepen our understanding of HIE and its applicability to mental health outcomes for various groups and generations. Nonetheless, significant differences by broad racial and ethnic groups suggest that mental health outcomes also differ by national origin groups as well.

Second, as with most generation studies, it is not possible to infer the exact magnitude of change in mental health scores since second generation respondents do not represent the parents of the third and higher generation. Although HILDA is a longitudinal study, the sample size is not large enough to measure intergenerational changes in mental health across families.

Third, despite the rich information in HILDA, it may be less representative of the Australian population than large scale census data due to lower response rates and panel attrition (Wilkins, 2015). Moreover, because of the longitudinal nature of the data, immigrants entering Australia after 2001 have a small chance of entering the sample so recently arrived immigrants may be less likely to be represented in the sample.

Fourth, this study shows an effect between gender and mental health that varies by generation. Future research should examine this interactive relationship to understand why the first generation do not encounter similar gender differences in mental health as their native-born counterparts.

## Conclusion

This study’s analysis of mental health among the first, second, and third and higher generations across several racial and ethnic backgrounds highlight some of the limitations of HIE. Diverging patterns of mental health by generation show the limited applicability of HIE for a broad range of outcomes and populations. In particular, HIE may be more reliable for understanding the first generation’s physical health outcomes, but not their mental health outcomes, partly because the factors predicting mental health differ from those predicting physical health.

This study suggests that relying solely on arguments of immigrant selection to understand mental health outcomes may be flawed. It is unclear whether it is easier to select individuals on their physical health relative to mental health in immigration policies or whether there are different predictors for the two health outcomes. Future research may examine the extent to which migrants are selective on physical health but not mental health or vice versa and whether selection criteria in immigration policies contribute to these differences. Nonetheless, the study’s findings have implications for immigration policymakers. If the goal of point-based policies is to select individuals who can easily integrate into society, lower mental health scores among immigrants suggest that selection criteria are not completely effective for predicting immigrants’ integration, disease prevention, and overall well-being in the host society.

Furthermore, differences in mental health scores by national origin and to a lesser extent generation status indicate different migration and adaptation processes, especially between English-speaking groups and others. This highlights the need for group-specific strategies and approaches to addressing mental health issues. Community-based research in different racial, ethnic, and immigrant populations may elucidate the mechanisms driving poor mental health and why similarity with host society members facilitates greater mental health outcomes. Likewise, greater in-depth case studies by specific national origin groups and nativity may clarify our understanding of how to reach certain populations and whether improving mental health outcomes may be related to providing higher quality care or greater access to services.

## Conflict of interest

I have no conflict of interest. I have no financial disclosures to report.

## Ethics approval

“Does the healthy immigrant effect apply to mental health? Examining the effects of immigrant generation and racial and ethnic background among Australian adults”.

Ethics approval is not required because I use secondary data analysis on a data set that available to all researchers working and living in Australia (Household, Income and Labour Dynamics in Australia). There is no identifiable information in the data.

## Figures and Tables

**Table A1:** Descriptive statistics of variables used in the analysis, by generation status.

	First generation	Second generation	Third and higher generation
Mental health score (0–100)	74.8	74.1	74.9
	(16.7)	(16.8)	(17.0)
			
Age	53.1	42.6	45.6
	(16.1)	(16.3)	(17.8)
			
Ethnicity			
Native-born Australian	–	–	100.0
English-speaking	41.8	52.5	–
European	15.8	30.3	–
North African and Middle Eastern	3.1	2.5	–
Asian	29.1	9.0	–
Other	10.1	5.8	–
			
Educational attainment			
HS degree or less	31.8	39.4	41.8
Diploma or certificate	30.5	32.8	34.0
Bachelor	19.2	16.6	14.1
Graduate/postgrad	18.5	11.3	10.1
			
Labor force status			
Employed	59.1	72.1	67.8
Unemployed	2.5	3.1	3.1
Not in labor force	38.4	24.8	29.0
			
Marital status			
Married	67.3	50.3	51.4
Widowed	5.6	2.9	4.0
Single	13.6	35.1	32.2
Divorced/separated	13.5	11.8	12.4
			
Remoteness			
Urban	82.5	74.5	60.0
Rural	16.4	24.3	38.5
Remote	1.2	1.2	1.5
			
Household income			
0–19,999	1.0	0.9	0.8
20,000–39,999	22.4	15.0	17.4
40,000–59,999	15.4	14.6	15.5
60,000–79,999	12.2	11.8	12.0
80,000–124,999	21.7	26.4	24.5
125,000+	27.4	31.4	29.8
Monolingual	56.1	88.6	99.3
	(49.6)	(31.8)	(8.2)
			
Neighborhood disadvantage			
Poorest	18.2	14.9	17.1
2nd Quintile	18.6	18.0	19.8
3rd Quintile	17.2	20.0	20.7
4th Quintile	20.6	24.0	22.7
Richest	25.4	23.2	19.7
Citizen	69.7	96.3	100.0
	(46.0)	(18.8)	(0)
			
Female	51.5	51.8	52.7
	(50.0)	(50.0)	(49.9)
			
Overweight	53.9	57.7	61.3
	(49.9)	(49.4)	(48.7)
			
N (observations)	10028	19337	56947

Note: standard deviation in parentheses for dichotomous variables.

## Appendix A

See Table A1.

## Footnotes

1 English-speaking refers to first and second generation individuals from English-speaking countries, including England, New Zealand, United States, Canada, etc.
